# Malaria in Pregnancy in Endemic Regions of Colombia: High Frequency of Asymptomatic and Peri-Urban Infections in Pregnant Women with Malaria

**DOI:** 10.1155/2020/2750258

**Published:** 2020-08-20

**Authors:** Ana-María Vásquez, Lina Zuluaga-Idárraga, Margarita Arboleda, Luz-Yáned Usuga, Carolina Gallego-Marin, Alvaro Lasso, Luisa Carbal, Juan-Gabriel Piñeros-Jiménez, Alberto Tobón-Castaño

**Affiliations:** ^1^Grupo Malaria, Facultad de Medicina, Universidad de Antioquia, Medellín-Antioquia, Colombia; ^2^Instituto Colombiano de Medicina Tropical (ICMT), Universidad CES, Carrera 98, #106-176 Apartadó-Antioquia, Colombia; ^3^Centro Internacional de Entrenamiento e Investigaciones Médicas (CIlDEIM), Calle 18, #122-135 Cali-Valle del Cauca, Colombia; ^4^Grupo de Investigación Salud y Ambiente, Facultad Nacional de Salud Pública, Universidad de Antioquia, Calle 62 #52-59. Medellín-Antioquia, Colombia

## Abstract

**Background:**

Malaria in pregnancy (MiP) has been associated with adverse pregnancy outcomes. There is limited information on MiP in low transmission regions as Colombia. This study aimed to describe the epidemiology of MiP through active surveillance of infections by microscopy and polymerase chain reaction (PCR).

**Methods:**

A cross-sectional study was conducted between May 2016 and January 2017 in five municipalities (Apartadó, Turbo, El Bagre, Quibdó, and Tumaco) in Colombia. Pregnant women self-presenting at health centers for antenatal care visits, seeking medical care for suspected malaria, or delivery, were enrolled. Diagnosis of *Plasmodium* spp was made in peripheral and placental blood samples by microscopy and PCR.

**Results:**

A total of 787 pregnant women were enrolled; plasmodial infection was diagnosed by microscopy in 4.2% (95% CI 2.8-5.6; 33/787) or by nPCR in 5.3% (95% CI 3.8-6.9; 42/787) in peripheral blood. Most of the infections were caused by *P. falciparum* (78.5%), and 46% were afebrile (asymptomatic). Women in the first and second trimester of pregnancy were more likely to be infected (aOR = 3.06, 95%CI = 1.6 − 5.8). To live in the urban/peri-urban area (aOR = 3.04, 95%CI = 1.4 − 6.56), to have a history of malaria during last year (aOR = 5.45, 95%IC = 2.16 − 13.75), and the infrequent bed net usage (aOR = 2.8, 95%CI = 1.31 − 5.97) were associated with the infection. Pregnant infected women had a higher risk of anaemia (aOR = 2.18, 95%CI = 1.15 − 4.12) and fever (aOR = 14.2, 95%CI = 6.89 − 29.8).

**Conclusion:**

The screening for malaria during antenatal care in endemic areas of Colombia is highly recommended due to the potential adverse effects of *Plasmodium* spp. infection in pregnancy and as an important activity for the surveillance of asymptomatic infections in the control of malaria.

## 1. Background

In the Americas region, where malaria transmission is low and unstable, 3 million pregnancies are at risk of infection with *Plasmodium falciparum* and 2.9 million with *Plasmodium vivax* [1]. Although the burden of malaria in pregnancy (MiP) is lower compared to high transmission areas, such as countries in sub-Saharan Africa, the infection is associated with adverse outcomes like maternal anaemia (MA), stillbirth, and low birth weight (LBW) caused by preterm birth or fetal growth restriction [2–4]. The epidemiology and clinical impact of MiP have been well documented in sub-Saharan Africa, where the prevalence of *P. falciparum* is higher in primigravidae than multigravidae women. During successive pregnancies, most of the infections became asymptomatic, due to antidisease immunity acquired from previous exposures [3, 4]. However, subclinical infections are also associated with MA [5, 6]. MiP has been poorly characterized in low transmission settings, mainly in Latin America, where primigravidae and multigravidae mothers are susceptible to infection due low levels of protective immunity to malaria [4].

A large proportion of asymptomatic infections are associated with low-density parasitaemias, which are difficult to detect by microscopy but are detected by molecular test as the polymerase chain reaction (PCR) [7]. Malaria infections in pregnancy, even asymptomatic and submicroscopic (defined as those infections detected by molecular test but not by microscopy), could be detrimental to mother, fetus, and newborn health; besides, these infections represent an import reservoir of gametocytes, which can contribute to sustaining malaria transmission [8].

The malaria transmission in Colombia is focal and variable throughout the endemic regions; the most affected municipalities are located at the Pacific coast, and the transmission is predominantly rural; although, 10% of the municipalities have a risk of peri-urban transmission [9]. There is little information on malaria infection in pregnancy; therefore, this study aimed to determine the frequency of *Plasmodium* spp infection during pregnancy in five malaria-endemic municipalities of Colombia by microscopy and PCR. We also described clinical characteristics of mothers and newborns and explored the association of socio-demographic and epidemiological factors to infection, in order to better characterize the epidemiology of MiP in the Americas.

## 2. Methods

### 2.1. Study Design and Study Areas

A cross-sectional study was conducted between May 2016 and September 2017, in five municipalities from three departments in Colombia, with different annual parasite index (API) [number of cases/1.000 habitants]. The study locations were selected based on the number of cases reported during previous years by the National Institute of Health in Colombia [10].

#### 2.1.1. Antioquia

The municipalities of Apartadó, Turbo, and El Bagre were included in the study; the transmission of malaria is in rural areas. Apartadó is located in the Urabá region (7.8828° N, 76.6247° W), API of 1.0 in 2017 (moderated risk), *P. vivax* caused 90% of cases. Turbo is located in the Urabá region (8.0926° N 76.7282° W), API of 1.2 in 2017 (moderated risk), *P. vivax* caused 78% of cases. El Bagre is located in the Bajo Cauca region (7.6014° N, 74.8053° W), API of 25.2 in 2016 (high risk), *P. vivax* caused 70% of cases.

#### 2.1.2. Chocó

The municipality of Quibdó is located in the Pacific region (5.6956° N, 76.6498° W), API of 100.8 in 2016 (high risk), *P. falciparum* caused 70% of cases. There is malaria transmission in rural and peri-urban settings.

#### 2.1.3. Nariño

Tumaco is located on the Pacific coast (1.7874° N, 78.7913° W), API of 16 in 2016 (high risk), *P. falciparum* caused 90% of cases. There is malaria transmission in rural and peri-urban settings.

### 2.2. Study Population

Pregnant women, self-presenting at health centers for antenatal care (ANC) visits, were seeking medical care for suspected malaria or delivery. Each participant was enrolled only once, in one of the two study groups (ANC or delivery).

### 2.3. Sample Size

Sample size was calculated for a proportion assuming an unknown population size, using the following equation: *n* = (*Z*2*pq*)/*d*2, where *n* is the sample size, *N* is the population size, *Z* is the confidence level, *p* is the expected proportion of MiP, *q* is the proportion of the population without MiP (1-p), and *d* is the margin of error. An expected prevalence of MiP of 4% was assumed, according to previous data from studies performed among different regions in South America [11–14]. A confidence interval of 95% and an error of 1.5% were used for the calculation; the minimum sample size calculated was 656 pregnant women. The sample size was distributed proportionally to the three main regions (Antioquia, Chocó, and Nariño) and ANC or at delivery groups.

To obtain the expected prevalence of MiP detected by microscopy, data from 10 reports of seven countries of Latin America were reviewed [11–20]. The highest frequency of MiP was observed in Colombia (10%), Peru (15%), and Venezuela (27%) in studies performed between 2002 and 2006; however, in the most recent four studies with a methodological design similar to the present study and conducted between 2004 and 2013, the mean of MiP prevalence was 4.7% and the median was 4% [11–14].

Inclusion criteria were the following: aged ≥15 years old, at any gestational age, resident in areas of the municipality with malaria transmission, and without previous antimalarial drug intake in the past 48 hours. When pregnant women resulted positive by microscopy, samples were taken before the treatment. Eligible pregnant women were enrolled after gave their written informed consent. All women diagnosed with malaria by microscopy were treated, according to the Colombian Ministry of Health recommendations [21].

### 2.4. Ethics

The study protocol and informed consent form were reviewed and approved by the Ethics Committee of the School of Medicine, Universidad de Antioquia (Record 005 of 31-03-2016). For women younger than 18 years old, informed assent was obtained from the parents or legal guardian.

### 2.5. Data and Sample Collection

Socio-demographic, reproductive history, and clinical information were recorded using an interview-based questionnaire. Axillary temperature was measured using a digital thermometer. A total of 8 mL of peripheral blood was collected by venipuncture using heparin tubes. When the participants were enrolled at delivery, new-born data were recorded, and 8 mL of placental blood was collected. Briefly, a small incision was made on the maternal side of the placenta, near the cord insertion area, placental blood accumulated at the place of insertion was collected by aspiration. Blood samples were processed immediately at the hospital's laboratory. Hemoglobin was measured by HemoCue AB® (Reference 201+, Sweden). Thick and thin blood smears were prepared from fresh blood, and PCR was performed from blood spotted on Whatman filter paper #3 (Fisher, Ref 1003-917).

### 2.6. Test Procedures

#### 2.6.1. Microscopy

A qualified malaria microscopist read Field-stained thick and thin blood slides, following the national guidelines [18]. A thick smear was considered negative if, after examination of 200 microscopic fields at 100x magnification, no parasites were found. For positive smears, parasite density was calculated by counting the number of parasites per 200 leukocytes (8,000 leukocytes/*μ*L, standard value) and was expressed as parasites/*μ*L (p/*μ*L). *P. falciparum* parasitaemia was calculated by counting ring forms, while *P. vivax* parasitaemia was calculated by counting all asexual forms (rings, trophozoites, and schizonts) and gametocytes. The gametocitaemia was calculated by counting the number of gametocytes of *P. falciparum* and *P. vivax* in 200 leukocytes and was expressed as gametocytes/*μ*L (g/*μ*L). As a quality control, a second reading was performed in all PCR positive samples and 10% of PCR negative samples.

#### 2.6.2. Nested PCR

DNA was extracted from half blood-spot filter (approximately 30 *μ*L of blood) using QIAamp DNA Mini Kit (Qiagen, Germany, Ref 51306), according to manufacturer's instructions. The nPCR was performed as a two-step procedure, using 2 *μ*L of DNA template and following the protocol described by Singh et al. 1999 [22], performing a first reaction to amplify the 18S ribosomal gene of Plasmodium genus (nest 1). Amplicons obtained from the first round of amplification were further processed in three separated reactions, using species-specific primers, to detect the 18S ribosomal gene of *P. falciparum*, *P. vivax*, and *Plasmodium malariae* (nest 2). Amplified products were revealed in a 1.5% agarose gel stained with GelRed ™ (Biotium, ref. 41003, United States). The sequences of primers used in the nPCR are shown in the supplementary table 1. The limit of detection of the nPCR used in the study was 1 parasite/*μ*L and was estimated using in vitro cultures of *P. falciparum*, preparing several dilutions with parasites densities ranging from 0.09 to 1000 parasites by microliter; DNA was extracted and PCR performed.

### 2.7. Definitions

Submicroscopic infections are *Plasmodium* spp infection detected by nPCR but not by microscopy. Febrile infections are plasmodial infections detected by any method in the presence of fever (axillary temperature ≥37.5°C) or history of fever in the last three days; this definition was used as a reference to symptomatic infections. Afebrile infections are infections detected by any method in pregnant women without fever or history of fever in the last three days; this definition was used as a reference to asymptomatic infections. Anaemia is define as hemoglobin level <11 g/dL, mild (10.0-10.9 g/dL), moderate (7.0-9.9 g/dL) and severe (≤6.9 g/dL), according to WHO guidelines [23]. LBW indicates a birth weight under 2500 gr. Small for gestational age (SGA) indicates neonates with a birth weight lower than the percentile ten for their age. Delivery preterm indicates neonates delivered before 37 weeks [4].

### 2.8. Statistical Analysis

The information was entered into an Access® database. Descriptive analysis was performed to summarize the baseline characteristics, using proportions and median (interquartile range—IQR). The proportion and its 95% confidence interval (CI) of infections detected by microscopy and nPCR were calculated. Chi-square test was used to compare the frequency of MiP and anaemia between ANC and delivery groups. The association of potential risk factors with MiP was analysed using a binary logistic regression. Crude and adjusted odds ratio (OR and aOR) and their 95% CI were calculated. The associated factors were selected based on the biological plausibility and a *p* value <0.25 in the bivariate analysis [24]. Additional analysis was carried out among malaria positive pregnant women comparing febrile and afebrile infection; chi-square test or Mann–Whitney *U* test was used. All analyses were carried out using SPSS-v25 software. *p* values <0.05 were considered significant.

## 3. Results

A total of 787 pregnant women were enrolled: 289 (37%) in Antioquia, 242 (31%) in Chocó, and 256 (32%) in Tumaco (Supplementary table 2). Overall, 58% (458/787) of participants were recruited during ANC and 42% (329/787) at delivery. The median age (IQR) of pregnant women was 23 years (20-28), and 39.4% were primigravidae. Most pregnant women were married (>80%) and had some level of formal education (63% secondary level, and 27.7% primary level). Study participants had attended a median (IQR) of four (2-6) ANC visits. Anaemia was found in 39.5% (302/786) and was more frequent at delivery compared to the ANC group (45.5% vs. 35.4%, *p* = 0.005). Other baseline characteristics are shown in Table 1.

### 3.1. Frequency of Infection among Pregnant Women

A total of 46/787 (5.8%; 95% CI 4.2-7.5) of pregnant women were infected by *Plasmodium* spp detected by nPCR (either in peripheral or placental blood compartments). The overall frequency of parasite presence in peripheral blood was 4.2% (95% CI 2.8-5.6; 33/787) by microscopy and 5.3% (95% CI 3.8-6.9; 42/787) by nPCR (Table 2). The frequency of infection was significantly higher in the ANC group than in the delivery group, when detected by microscopy (7.2% vs. 0%, *p* < 0.001), and nPCR (8.1% vs. 4.5%, *p* < 0.001). All peripheral blood samples positives for *Plasmodium* infection by microscopy were also positive by nPCR, and 9 (1.2%) with negative smear were positive by nPCR (i.e., submicroscopic infections). Most infections were caused by *P. falciparum* (79%, 33/42).

The median of parasite density by microscopy (*n* = 33) was 2480 parasites/*μ*L (IQR 1130-4018); 1952 parasites/*μ*L for *P. falciparum* mono-infection (*n* = 26) and 6180 parasites/*μ*L for *P. vivax* mono-infection (*n* = 6). A total of 40.7% (11/27) of pregnant women with microscopic infection by *P. falciparum* had gametocytes with a median (IQR) of 32 gametocytes/*μ*L (24-88), while a total of 87.5% (7/8) of pregnant women with microscopic infection by *P. vivax* had gametocytes with a median (IQR) of 328 gametocytes/*μ*L (120-1000).

Placental infection was detected in 2 out of 239 (0.6%; 95% CI 0-1.4) of placental samples using microscopy and 9 out of 329 (2.7%; 95% CI 1-4.5) using nPCR, from those eight were caused by *P. falciparum* and one by *P. vivax*. Among nine pregnant women with placental infection by nPCR, five were also positive in peripheral blood and four only in the placenta.

### 3.2. Socio-Demographic and Obstetric Characteristics among Infected Pregnant Women

Maternal age, marital status, education level, socioeconomic status, and occupational exposure to mosquito bites were similar between infected and noninfected pregnant women (Table 3). Most of the infected pregnant women were living in the urban/peri-urban area of the municipalities, at the lower socioeconomic status, and around half of them had water bodies around their homes. Living in urban/peri-urban was associated with being infected (Tables 3, OR 3.79, 95% CI 1.80-7.99).

The proportion of primigravidae was similar between infected and noninfected pregnant women (39.1% vs. 38.3%). Gestational age and being in the first and second trimester were associated with infection (Table 3). Among infected women, 15% (7/46) self-reported history of previous malaria episodes during last year was compared to 4.3% (32/741) in uninfected women (OR 3.97, 95%CI = 1.6 − 9.6). The proportion of participants with self-reported malaria during the current pregnancy was also higher in infected than uninfected women (OR 12.7, 95%CI = 5.7 − 28.1). Related to prevention activities, the usage of bed nets sometimes was associated with infection (Table 3).

### 3.3. Anaemia, Fever, and Birth Outcomes

Infected pregnant women had lower hemoglobin levels than uninfected women (Table 4). Infection was associated with two-fold increased odds of having anaemia (aOR = 2.18, 95%CI = 1.15 − 4.12) and fourteen-fold increased odds of having fever or history of fever (aOR = 14.2, 95%CI = 6.89 − 29.8) (Table 5). The median of the birth weight was lower in the neonates from infected pregnant women compared to no-infected (2500 g vs. 3200 g, *p* = 0.009), and the proportion of neonates small for gestational age (SGA) was higher in the infected pregnant women.

### 3.4. Factors Associated with *Plasmodium* spp Infection in Pregnancy

Being in the first or second trimester (aOR = 3.06, 95%CI = 1.6 − 5.8), to live in the urban/peri-urban area (aOR = 3.04, 95%CI = 1.4 − 6.56), to have history of malaria during last year (aOR = 5.45, 95%CI = 2.16 − 13.75), history of malaria in a cohabitant (aOR = 3.22, 95%CI = 1.46 − 7.12), and the infrequent usage of bed net (aOR = 2.8, 95%CI = 1.31 − 5.97) were associated with infection (Table 5).

### 3.5. Afebrile Infections

The frequency of afebrile (asymptomatic) infections was 46% (21/46). The characteristics of afebrile and febrile infections are shown in Table 6. The median parasite density was significantly lower in afebrile infected pregnant women than in febrile (1410 vs. 3235 parasites/*μ*L); also, the proportion of submicroscopic infections were higher in afebrile as compared with febrile women (Table 6).

## 4. Discussion

The frequency of malaria among pregnant women attending ANCs and delivering at the study site hospitals was 4.2% detected by microscopy. The infection during pregnancy was associated with negative consequences for maternal health like anaemia that may have a negative impact on neonatal health. Importantly, most asymptomatic infections were undetected with microscopic examination, which is the routine test used to diagnose malaria in Colombia. Additionally, 40% of *P. falciparum* infections had gametocytes, and most of them were asymptomatic.

Less is known about the burden and impact of infection in low transmission scenarios, particularly in the Americas where *P. vivax* infections are most frequent. In this region, there was an incidence of MiP of 94 cases/100.000 pregnant women in 2014, which varies among countries [25]. The prevalence of infection measured by microscopy ranged from 0.3% to 27% in countries from South America, with variations between the periods of time [11–14, 17–20, 26–29]. The highest prevalence was observed in Colombia (13%) and Venezuela (27%) during 2005-2006 [19, 20]. However, recent studies in Brazil and Colombia, including the current survey, indicated a decrease in the prevalence of infection (less than 5%), that correlates with the general reduction of cases in the region during the last years, and which could be associated to several malaria preventive and control measures adopted [27].

In Colombia, MiP has been mainly studied in the north-west region of the country during 2005-2011, detecting a prevalence ranged between 1 and 13%; most of the cases were caused by *P. vivax* [12, 19, 28]. In those studies, MiP was associated with complicated malaria [30], low birth weight, and prematurity [31]. There are no data of MiP in other endemic areas in Colombia, as the Pacific region, that nowadays reports the majority of malaria cases in the country. This study reported a frequency of infection of 8.7% in Quibdó-Chocó and 7.4% in Tumaco-Nariño detected by PCR (supplementary table 2). In both localities, most of the infected pregnant women were living in the urban/peri-urban areas (90% and 89.5%, respectively). Malaria transmission in the urban and peri-urban areas on the Pacific coast region has been reported; although, the epidemiological and entomological conditions that contribute to the urban presence of malaria remain unexplored. It has been suggested that population movements, including infected individuals from rural to urban areas, contribute to the introduction and maintenance of Plasmodium transmission in these areas [32].

Similar to the current findings, recent studies indicated that microscopy infections by *P. falciparum* and *P. vivax* were associated with MA [27] as well as with a reduction in hemoglobin levels [29, 33], reduction in birth weight [29, 33], a high risk for LBW, SGA, and preterm birth [27, 33]. In this study, it was observed that infected pregnant women had a 2-fold higher risk of anaemia compared to noninfected women. It is well known that MA has a negative impact not only on maternal mortality and morbidity but also on neonatal health [4]. It is important to consider that there may be other factors associated with anaemia in this population, such as iron and/or folate deficiency and hookworm infection, which are prevalent in these settings [34–36].

A significant proportion of infected pregnant women carries subpatent parasitaemias that are not detected by routine test [11, 12, 14, 27, 28]. The clinical impact of asymptomatic and subpatent infections remains understood, and their importance needs to be investigated [3]. From the few studies in Latin America, there is no correlation between subpatent infections and poor pregnancy outcomes [27, 28].

In this study, it was observed that previous malaria episodes during last year and the infrequent usage of bed nets were risk factors for MiP. Previous episodes of malaria may reflect a continuous vector exposure explained by residence conditions and preventive measured adopted, among others. In a previous study conducted in Antioquia and Chocó, it was reported that 86% and 93% of participants had at least one mosquito net, respectively; however, 72-73% used it daily [37]. Together, these findings highlight the role of bed nets preventing vector exposure, and suggest the need for strategies to improve their usage and care.

There was no statistical association between gravidity and infection. Studies from high transmission areas in Africa have shown a gravidity-dependent susceptibility to malaria, which has been attributed to the acquisition of antibodies to pregnancy-associated *P. falciparum* parasite strains [3]. Similar to reported in Asia, regardless of the number of previous pregnancies, most pregnant women from low transmission settings have little immunity to malaria, and every single infection may have adverse consequences on mothers, fetuses, and neonates [38].

Women in the first and second trimester of pregnancy were more likely to be infected. At the end of pregnancy, a reduction of microscopic infections and an increase of subpatent infections have been reported; this could be explained because of previous infections treated, and possible acquisition of immunity as the pregnancy progressed [39]. There is little knowledge about the effect of malaria during the first half of pregnancy, but it is well known that, in this period, the development of the placental circulation takes place. Recently, it was reported that placentas from pregnant women who had an infection before of 15th week of gestation showed morphological changes compatible with an impairment of vascular development, which was associated with RCIU and BPN [40].

Most of the studies have used the absence of fever as a standard definition of asymptomatic infection, especially in pregnant women, due to the fact that the signs and symptoms associated with malaria are common during pregnancy. Most of the malarial clinical symptoms had low positive predictive values (less than 40%), and only the fever or history of fever during last 72 hours has been associated with infection [41]. In this study, it was observed that 46% of infected pregnant women were afebrile, suggesting the importance of malaria-screening in all pregnant women living in endemic areas, regardless the presence of symptoms, even more, when they often have gametocytes. Untreated infections in pregnant women may represent a reservoir of malaria that contributes to sustaining transmission.

This study had some limitations. First, the cross-sectional design that provides an estimate of the burden of the infection in a single period. A longitudinal follow-up of pregnant women would have allowed us better quantify the burden and consequences of infection. Second, the sample size was not calculated for each geographic area; thus, the findings of this study should be interpreted with caution. Third, this study was focalized on women attending health facilities and did not evaluate women who did not attend the ANC or had community delivery. Fourth, the evaluation of gestational age by ultrasound was not available in most of cases, and the Ballards method was used, which is not as reliable as ultrasound is. Finally, the number of infected pregnant women detected was modest, which limited the power to detect associations.

## 5. Conclusions

This study shows a low frequency of pregnant woman infected by *Plasmodium* across study sites but suggests that asymptomatic and submicroscopic infections are common. The submicroscopic infections were higher in some areas, like Tumaco (south Pacific Coast), which may have implications for the diagnosis of malaria with the currently used test. Routine screening of malaria during ANC visits is recommended due to the fact that infection is associated with maternal anaemia, which may have a negative impact on neonatal and infant health. Furthermore, the high proportion of afebrile infections supports the importance of the screening in all pregnant women regardless of the presence of symptoms. Also, we found a significant proportion of infected pregnant women that carry gametocytes, and their role as parasite reservoir must be considered in surveillance activities towards malaria elimination.

## Figures and Tables

**Table 1:** Baseline characteristics of pregnant women enrolled.

	ANC (*n* = 458)	Delivery (*n* = 329)	Total (*n* = 787)
Age (years): median (IQR)	23 (19.0-28.0)	24.0 (21.0-28.0)	23 (20.0-28.0)
<20 years old: *n* (%)	159 (34.7)	71 (21.6)	230 (29.2)
Rural residence: *n* (%)	227 (51.6)	165 (50.6)	392 (51.2)
Head of household: *n* (%)	45 (10.3)	40 (12.4)	85 (11.2)
Gestational age (weeks): median (IQR)	20 (13.0-27.0)	38 (37.0-39.0)	30 (17.0-38.0)
Gravidity: median (IQR)	1 (0.0-2.0)	1 (0.0-1.0)	1 (0.0-2.0)
Primigravidae: *n* (%)	153 (33.4)	149 (48.4)	302 (39.4)
# ANC visits: median (IQR)	3 (1.0-4.0)	6 (5.0-7.0)	4 (2.0-6.0)
Hemoglobin (g/dL): median (IQR)	11.5 (10.3-12.1)	11.4 (9.1-13.0)	11.5 (10.0-12.4)
Anaemia: *n* (%)	162 (35.4)	140 (45.5)	302 (39.5)
Fever/history of fever 48 h: *n* (%)	34 (8.5)	28 (8.8)	62 (8.7)
Malaria history (self-report): *n* (%)			
Malaria during last year	22 (4.8)	17 (5.2)	39 (5.0)
Malaria in the current pregnancy	20 (4.4)	11 (3.6)	32 (4.1)
Use of bed net: *n* (%)			
Always	226 (49.8)	137 (44.8)	363 (47.8)
Most of the time	49 (10.8)	34 (11.1)	83 (10.9)
Some times	59 (13)	94 (30.7)	153 (20.1)
Never	120 (26.4)	41 (13.4)	161 (21.2)

ANC: antenatal care; IQR: interquartile range; *n*: sample size.

**Table 2:** Frequency of infection among pregnant women attending antenatal clinics and delivery units.

	Infection in peripheral blood			Infection in placental blood
	Total	ANC	Delivery	
	**(** **n** = 787**)**	**(** **n** = 458**)**	**(** **n** = 329**)**	**(** **n** = 329**)**
Microscopy: *n* (%)	33 (4.2)	33 (7.2)	0 (0)	2 (0.6)
*P. falciparum*	26 (3.3)	26 (5.7)	0 (0)	2 (0.6)
*P. vivax*	6 (0.8)	6 (1.3)	0 (0)	0 (0)
Mixed P.F and P.V	1 (0.1)	1 (0.2)	0 (0)	0 (0)
nPCR: *n* (%)	42 (5.3)^∗^	37 (8.1)	5 (1.5)	9 (2.7)
*P. falciparum*	33 (4.2)	28 (6.1)	5 (1.5)	8 (2.4)
*P. vivax*	6 (0.8)	6 (1.3)	0 (0)	1 (0.3)
*P. malarie*	1 (0.1)	1 (0.2)	0 (0)	0 (0)
Mixed P.F and P.V	2 (0.2)	2 (0.4)	0 (0)	0 (0)
Submicroscopic infections: *n* (%)	9 (1.1)	4 (0.9)	5 (1.5)	7 (2.1)
Parasitaemia				
Median		2480		180
(IQR)		(1130-4018)		(n.a)

IQR: interquartile range; SD: standard deviation; *n* (sample size). ^∗^A total of 46 infected pregnant women were detected; 42 were positive in peripheral blood by nPCR; and 9 were positive in placental blood, from those 5 were also positive in peripheral blood.

**Table 3:** Socio-demographic and obstetric characteristics of infected pregnant women.

	Infected (*n* = 46)	No-infected (*n* = 741)	OR^∗∗^	95% IC
Socio-demographic characteristics^∗^				
Age (years)	25.5 (19.7-28.2)	23 (20.0-28.0)	1.03	0.98-1.08
<20 years old: *n* (%)	13 (28.3)	217 (29.3)	0.95	0.49-1.84
Head of household: *n* (%)	38 (82.6)	615 (84.8)	1.07	0.41-2.81
# people/house	4.5 (4.0-5.0)	4 (3.0-6.0)	0.916	0.73-1.15
Socioeconomic stratum ≤1: *n* (%)	46 (100)	675 (98.8)	ND	ND
Occupational exposure to mosquito bite: *n* (%)	2 (4.4)	17 (2.4)	1.87	0.42-8.36
Residence area: *n* (%)				
Rural	9 (20.0)	351 (48.7)	1	
Urban and peri-urban	36 (80.0)	370 (51.3)	**3.79**	**1.80-7.99**
Water service: *n* (%)	23 (50.0)	268 (36.3)	0.57	0.31-1.03
Water bodies around the home: *n* (%)	25 (54.3)	398 (54.5)	0.99	0.55-1.81
Obstetric characteristics				
Gestational age (weeks)	20 (16.00-32.75)	32 (18.0-38.0)	**0.96**	**0.94-0.99**
Trimester: *n* (%)				
First	9 (19.6)	101 (13.7)	**2.43**	**1.04-5.65**
Second	21 (45.7)	201 (27.2)	**2.85**	**1.45-5.57**
Third	16 (34.8)	436 (59.1)	1	
# previous pregnancies	1 (0.0-2.0)	1 (0.0-2.0)	1.01	0.84-1.23
Primigravidae: *n* (%)	18 (39.1)	284 (38.3)	1.03	0.56-1.90
# ANC visits	3 (2.0-5.0)	4 (2.0-6.0)	**0.85**	**0.73-0.99**
Malaria related characteristics (self-report)				
Living years in the area	20 (6.0-27.2)	19 (5.0-23.0)	1.02	0.99-1.05
Malaria during last year: *n* (%)	7 (15.2)	32 (4.3)	**3.97**	**1.65-9.56**
Malaria in the current pregnancy: *n* (%)	12 (26.1)	20 (2.7)	**12.71**	**5.74-28.11**
Malaria in a cohabitant (last 6 months): *n* (%)	15 (34.9)	95 (13.5)	**3.40**	**1.75-6.60**
Use of bed net: *n* (%)				
Always	16 (34.8)	362 (49.3)		
Most of the time	8 (17.4)	75 (10.2)	**2.41**	**1.00-5.84**
Some times	16 (34.8)	140 (19)	**2.57**	**1.26-5.31**
Never	6 (13)	158 (21.5)	0.86	0.33-2.24

IQR: interquartile range; CI95: confidence Interval of 95%; *n* (sample size). ^∗^Binary logistic regression. Maternal infections were considered if nPCR was positive by *Plasmodium spp* in peripheral blood or placental blood (at delivery).

**Table 4:** Clinical characteristics of infected pregnant women and birth outcomes.

Clinical characteristics	Infected (*n* = 46)	No-infected (*n* = 741)	OR^∗^	IC95
Axillary temperature (°C)	37 (36.6-37.6)	36.7 (36.3-37)	**5.06**	**2.85-8.98**
Fever/history of fever 48 h: *n* (%)	25 (54.3)	42 (5.7)	**12.77**	**6.50-25.11**
Hemoglobin (g/dL)	10.6 (9.35-12.0)	11.6 (10.0-12.5)	8.83	0.70-0.99
Anaemia: *n* (%)	25 (55.6)	282 (31.1)	**2.03**	**1.11-3.73**
Category of anaemia: *n* (%)				
Severe	0	4 (1.4)	ND	ND
Moderate	8 (32.0)	75 (26.6)	1.27	0.53-3.07
Mild	17 (68.0)	203 (72.0)	1	
Neonatal health (*n* = 329)	**n** = 9	**n** = 320		
Gestational age at delivery	38 (37.0-38.0)	38 (37.0-39.0)		
Preterm birth: *n* (%)	0 (0)	29 (9.2)		
Weight (g)^ⱡ^	2500 (2800-2930)	3200 (2845-3500)		
LBW: *n* (%)	2 (22.2)	19 (6.0)		
SGA: *n* (%)	2 (22.2)	15 (4.8)		

IQR: interquartile range; CI95: confidence interval of 95%; *n*: sample size; OR: odds ratio; LBW: low birth weight; SGA: small for gestational age. ^∗^Quantitative data are described in median (IQR) and qualitative data in number (%). ^∗∗^Binary logistic regression. ^ⱡ^Man *U* test (*p* = 0.009). Maternal infections were considered if nPCR was positive by *Plasmodium* spp in peripheral blood or placental blood (at delivery).

**Table 5:** Logistic regression analysis of associated factors to *Plasmodium* spp infection among pregnant women.

	*n*	Infected: *n* (%/*n*)	aOR^∗^	95% CI
Trimester				
First and second	332	30 (9.0)	**3.06**	**1.61-5.80**
Third	452	16 (3.5)	1	
Gravidae				
Primigravidae	302	18 (5.9)	1.01	0.54-1.89
Multigravidae	485	28 (5.8)	1	
# ANC visits			1.05	0.84-1.31
Residence area				
Rural	392	9 (2.3)	1	
Urban	395	36 (9.1)	**3.04**	**1.41-6.56**
Living years in the area			1.03	1.00-1.06
Malaria during last year (self-report)				
Yes	39	7 (17.9)	**5.45**	**2.16-13.75**
No	748	39 (5.2)	1	
Malaria in a cohabitant in the last six months				
Yes	110	15 (13.6)	**3.22**	**1.46-7.12**
No	635	28 (4.4)	1	
Use of net: *n* (%)				
Always	378	16 (4.2)	1	
Most of the time	83	8 (9.6)	2.04	0.83-5.05
Some times	156	16 (10.2)	**2.80**	**1.31-5.97**
Never	164	6 (3.6)	0.95	0.36-2.51
Fever/history of fever 48 h				
Yes	62	20 (32.2)	**14.26**	**6.89-29.81**
No	725	26 (3.6)	1	
Anaemia				
Yes	307	25 (8.1)	**2.18**	**1.15-4.12**
No	480	21 (4.4)	1	

aOR: adjusted OR; CI95: confidence interval of 95%; aOR: adjusted odds ratio. ^∗^Binary logistic regression; adjusted by malaria during last year (self-report), trimester, and living years in area. Maternal infections were considered if nPCR was positive by *Plasmodium* spp in peripheral blood or placental blood (at delivery).

**Table 6:** Description of afebrile infections among infected pregnant women.

	Febrile (symptomatic) *n* = 25	Afebrile (asymptomatic) *n* = 21	*p* value^∗^
Positive microscopy^+^: *n* (%)	25 (100%)	8 (38%)	<0.001
Submicroscopic infection^+^: *n* (%)	0 (0)	13 (62)	
Parasitaemia: median (IQR)	3235 (1200-5880)	1410 (545-1992)	0.049
Parasitaemia <2000 p/*μ*L: *n* (%)	8/25 (32)	6/8 (75)	0.032
Parasitaemia >2000 p/*μ*L: *n* (%)	17/25 (68)	2/8 (25)	
Symptoms: *n* (%)			
Sweating	25 (100)	6 (29)	<0.001
Chills	24 (96)	4 (19)	<0.001
Headache	24 (96)	5 (24)	<0.001
Myalgia/arthralgia	19 (76)	9 (42.9)	0.022
Asthenia	16 (64)	7 (33)	0.038
Vomit	15 (60)	0	<0.001
Nauseas	10 (40)	5 (24)	0.24
Dizziness	14 (56)	2 (9.5)	0.001
Abdominal pain	11 (44)	8 (38.1)	0.68
Diarrhea	7 (28)	1 (5)	0.22

IQR: interquartile range. ^∗^Proportions were compared with chi^2^; medians were compared with Mann–Whitney *U* test. ^+^In peripheral blood.

## Data Availability

The datasets used and/or analysed during the current study are available from the corresponding author on reasonable request.

## Abbreviations

aOR: Adjusted odds ratio
ANC: Antenatal care
API: Annual parasite index
CI: Confidence interval
DNA: Deoxyribonucleic acid
IQR: Interquartile range
ITNs: Insecticide-treated bed nets
LBW: Low birth weight
MA: Maternal anaemia
MiP: Malaria in pregnancy
OR: Odds ratio
p/*μ*L: Parasite/*μ*L
*P. falciparum*: *Plasmodium falciparum*
*Plasmodium* spp: Plasmodium species
*P. vivax*: *Plasmodium vivax*
PCR: Polymerase chain reaction
nPCR: Nested polymerase chain reaction
*spp*: species
RDT: rapid diagnostic test
SGA: Small for gestational age
UV: Ultraviolet.

## Supplementary Materials

Supplementary materials Supplementary Table 1: sequence of the primers used in the nested polymerase chain reaction (nPCR) assay. Supplementary Table 2: frequency of Plasmodium spp infection and anaemia among the municipalities.

## References

[1] Dellicour S., Tatem A. J., Guerra C. A., Snow R. W., ter Kuile F. O. (2010). Quantifying the number of pregnancies at risk of malaria in 2007: a demographic study. *PLoS Medicine*.

[2] Yanow S. K., Gavina K., Gnidehou S., Maestre A. (2016). Impact of malaria in pregnancy as Latin America approaches elimination. *Trends in Parasitology*.

[3] Rogerson S. J., Desai M., Mayor A., Sicuri E., Taylor S. M., van Eijk A. M. (2018). Burden, pathology, and costs of malaria in pregnancy: new developments for an old problem. *The Lancet Infectious Diseases*.

[4] Desai M., ter Kuile F. O., Nosten F. (2007). Epidemiology and burden of malaria in pregnancy. *The Lancet Infectious Diseases*.

[5] Matangila J. R., Lufuluabo J., Ibalanky A. L., Inocêncio da Luz R. A., Lutumba P., Van Geertruyden J.-P. (2014). Asymptomatic Plasmodium falciparum infection is associated with anaemia in pregnancy and can be more cost-effectively detected by rapid diagnostic test than by microscopy in Kinshasa, Democratic Republic of the Congo. *Malaria Journal*.

[6] Nega D., Dana D., Tefera T., Eshetu T. (2015). Anemia associated with asymptomatic malaria among pregnant women in the rural surroundings of Arba Minch Town, South Ethiopia. *BMC Research Notes*.

[7] Okell L. C., Ghani A. C., Lyons E., Drakeley C. J. (2009). Submicroscopic infection in Plasmodium falciparum-endemic populations: a systematic review and meta-analysis. *The Journal of Infectious Diseases*.

[8] Boudová S., Cohee L. M., Kalilani-Phiri L. (2014). Pregnant women are a reservoir of malaria transmission in Blantyre, Malawi. *Malaria Journal*.

[9] Rodríguez J. C. P., Uribe G. Á., Araújo R. M., Narváez P. C., Valencia S. H. (2011). Epidemiology and control of malaria in Colombia. *Memórias do Instituto Oswaldo Cruz*.

[10] Instituto Nacional de Salud (INS) (2018). *Boletín epidemiológico*.

[11] Davison B. B., Branch O. L. H., Gamboa D., Parekh F. K., Hernandez J. (2010). Placental histopathologic changes associated with subclinical malaria infection and its impact on the fetal environment. *The American Journal of Tropical Medicine and Hygiene*.

[12] Agudelo O., Arango E., Maestre A., Carmona-Fonseca J. (2013). Prevalence of gestational, placental and congenital malaria in north-west Colombia. *Malaria Journal*.

[13] Brutus L., Santalla J., Schneider D., Avila J. C., Deloron P. (2013). Plasmodium vivax malaria during pregnancy, Bolivia. *Emerging Infectious Diseases*.

[14] Hristov A. D., Sanchez M. C. A., Ferreira J. J. B. (2014). Malaria in pregnant women living in areas of low transmission on the southeast Brazilian Coast: molecular diagnosis and humoural immunity profile. *Memórias do Instituto Oswaldo Cruz*.

[15] Rivera J., Rivera R. L., Dubón J. M., Reyes M. E. (1993). Efecto de la Malaria por Plasmodium vivax en la Salud Perinatal. *Revista Honduras Pediátrica*.

[16] Jarude R., Trindade R., Tavares-Neto J. (2003). Malária em grávidas de uma maternidade pública de Rio Branco (Acre, Brasil). *Revista Brasileira de Ginecologia e Obstetrícia*.

[17] Parekh F. K., Casapia W. M., Krogstad D. J., Branch O. H., Hernandez J. N. (2007). Prevalence and risk of Plasmodium falciparum and P. vivax malaria among pregnant women living in the hypoendemic communities of the Peruvian Amazon. *The American Journal of Tropical Medicine and Hygiene*.

[18] Asayag R. (2008). Malaria in pregnant women between March 2002 and July 2003: experience in Hospital Regional de Loreto, Peru. *Acta Médica Peruana*.

[19] Carmona-Fonseca J., Maestre-B A. (2009). Incidencia de las malarias gestacional, congénita y placentaria en Urabá (Antioquia, Colombia), 2005-2007. *Revista Colombiana de Obstetricia y Ginecología*.

[20] Gómez E., López E., Ache A. (2009). Malaria and pregnancy. San Isidro parish, municipality Sifontes, state of Bolívar, Venezuela, 2005-2006. *Investigación Clínica*.

[21] Padilla J. C., Montoya R. (2011). Guía de Atención Clínica de Malaria. *Infectio*.

[22] Snounou G., Abdullah M. S., Rahman H. A., Singh B., Bobogare A., Cox-Singh J. (1999). A genus- and species-specific nested polymerase chain reaction malaria detection assay for epidemiologic studies. *The American Journal of Tropical Medicine and Hygiene*.

[23] World Health Organization (WHO) (2011). *Haemoglobin concentrations for the diagnosis of anaemia and assessment of severity*.

[24] Hosmer D. J., SJ L, JWa S. Model-building strategies and methods for logistic regression. *Applied logistic regression*.

[25] Pan American Health Organization (PAHO) (2017). *Report on the Situation of Malaria in the Americas 2014*.

[26] Espinoza E., Hidalgo L., Chedraui P. (2005). The effect of malarial infection on maternal-fetal outcome in Ecuador. *The Journal of Maternal-Fetal & Neonatal Medicine*.

[27] Bardají A., Martínez-Espinosa F. E., Arévalo-Herrera M. (2017). Burden and impact of Plasmodium vivax in pregnancy: a multi-centre prospective observational study. *PLoS Neglected Tropical Diseases*.

[28] Gavina K., Gnidehou S., Arango E. (2018). Clinical Outcomes of Submicroscopic Infections and Correlates of Protection of VAR2CSA Antibodies in a Longitudinal Study of Pregnant Women in Colombia. *Infection and Immunity*.

[29] Pincelli A., Neves P. A. R., Lourenço B. H. (2018). The hidden burden of Plasmodium vivax malaria in pregnancy in the Amazon: an observational study in northwestern Brazil. *The American Journal of Tropical Medicine and Hygiene*.

[30] Piñeros J. G., Blair S., Álvarez G., Tobon-Castaño A., Portilla C. (2013). Maternal Clinical Findings in Malaria in Pregnancy in a Region of Northwestern Colombia. *The American Journal of Tropical Medicine and Hygiene*.

[31] Tobón-Castaño A., Solano M. A., Sánchez L. G. Á., Trujillo S. B. (2011). Retardo no crescimento intrauterino, baixo peso ao nascer e prematuridade em recém-nascidos de grávidas com malária, na Colômbia. *Revista da Sociedade Brasileira de Medicina Tropical*.

[32] Padilla J. C., Chaparro P. E., Molina K., Arevalo-Herrera M., Herrera S. (2015). Is there malaria transmission in urban settings in Colombia?. *Malaria Journal*.

[33] Dombrowski J. G., Souza R. M. d., Silva N. R. M. (2018). Malaria during pregnancy and newborn outcome in an unstable transmission area in Brazil: A population-based record linkage study. *PLOS ONE*.

[34] Valencia C. A., Fernández J. A., Cucunubá Z. M., Reyes P., López M. C., Duque S. (2010). Correlation between malaria incidence and prevalence of soil-transmitted helminths in Colombia: an ecologic evaluation. *Biomédica*.

[35] Ramírez-Vélez R., González-Ruíz K., Correa-Bautista J., Martínez-Torres J., Meneses-Echávez J. F., Rincon-Pabon D. (2014). Ferritin levels in pregnant Colombian women. *Nutrición Hospitalaria*.

[36] Ramírez-Vélez R., Correa-Bautista J. E., Martínez-Torres J., Meneses-Echávez J. F., Lobelo F. (2016). Vitamin B12 concentrations in pregnant Colombian women: analysis of nationwide data 2010. *BMC Pregnancy and Childbirth*.

[37] Londoño D. A. C., Álvarez O. N., Osorio L., Jiménez J. G. P., Uribe G. L. R. (2018). Conocimientos sobre malaria y prácticas de uso de mosquiteros con insecticidas de larga duración en dos departamentos de Colombia. *Revista Peruana de Medicina Experimental y Salud Pública*.

[38] Rijken M. J., McGready R., Boel M. E. (2012). Malaria in pregnancy in the Asia-Pacific region. *The Lancet Infectious Diseases*.

[39] Williams J. E., Cairns M., Njie F. (2016). The performance of a rapid diagnostic test in detecting malaria infection in pregnant women and the impact of missed infections. *Clinical Infectious Diseases*.

[40] Moeller S. L., Nyengaard J. R., Larsen L. G. (2019). Malaria in early pregnancy and the development of the placental vasculature. *The Journal of Infectious Diseases*.

[41] Tahita M. C., Tinto H., Menten J. (2013). Clinical signs and symptoms cannot reliably predict Plasmodium falciparum malaria infection in pregnant women living in an area of high seasonal transmission. *Malaria Journal*.

