# Supplementary material for: Malaria in Pregnancy in Endemic Regions of Colombia: High Frequency of Asymptomatic and Peri-Urban Infections in Pregnant Women with Malaria
Source: Infect Dis Obstet Gynecol. 2020 Aug 20;2020:2750258. doi: 10.1155/2020/2750258 (PMC7455813; doi:10.1155/2020/2750258)
Supplement: Supplementary materials — Supplementary Table 1: sequence of the primers used in the nested polymerase chain reaction (nPCR) assay. Supplementary Table 2: frequency of Plasmodium spp infection and anaemia among the municipalities. [file 2750258.f1.docx]

**Supplementary table 1. Sequence of the primers used in the nested polymerase chain reaction (nPCR) assay.**

| **Nest 1 genus-specific primers** |
| --- |
| PLU1 5" TCA AAG ATT AAG CCA TGC AAG TGA 3"´ |
| PLU5 5" CCT GTT GTT GCC TTA AAC TTC 3" |
| **Nest 2 species-specific primers** |
| FAL1 5´-TTA AAC TGG TTT GGG AAA ACC AAA TAT ATT -3´ |
| FAL2 5´- ACA CAA TGA ACT CAA TCA TGA CTA CCC GTC-3´ |
| VIV1 5´- CGC TTC TAG CTT AAT CCA CAT AAC TGA TAC-3´ |
| VIV2 5´- ACT TCC AAG CCG AAG CAA AGA AAG TCC TTA-3´ |
| MAL1 5´-ATA ACA TAG TTG TAC GTT AAG AAT AAC CGC -3´ |
| MAL2 5´ - AAA ATT CCC ATG CAT AAA AAA TTA TAC AAA -3´ |

**Supplementary table 2. Frequency of *Plasmodium* spp infection and anemia among the municipalities.**

|  | **ANTIOQUIA**  (Apartadó, El bagre, Turbo)  **(n=289)** | **CHOCÓ**  (Quibdó)  **(n=242)** | **NARIÑO**  (Tumaco)  **(n=256)** |
| --- | --- | --- | --- |
| Total of infected pregnant women: n (%) | 6 (2.1) | 21 (8.7) | 19 (7.4) |
| Proportion of asymptomatic infection: n/N (%) | 2/6 (33.3) | 6/21 (29.0) | 13/19 (68.4) |
|  |  |  |  |
| **Proportion of *Plasmodium* spp. n/N (%)** |  |  |  |
| *P. falciparum* | 2 (33.3) | 17 (81.0) | 17 (89.4) |
| *P. vivax* | 4 (66.7) | 2 (9.5) | 1 (5.3) |
| *P. malarie* | 0 (0.0) | 0 (0.0) | 1 (5.3) |
| *Mixed Pf, Pv* | 0 (0.0) | 2 (9.5) | 0 (0.0) |
|  |  |  |  |
| **Peripheral parasitaemia: n (%)** |  |  |  |
| Detected by PCR | 6 (2.1) | 18 (7.4) | 18 (7.0) |
| Detected by microscopy | 5 (1.7) | 18 (7.4) | 10 (3.9) |
|  |  |  |  |
| **Residence of infected pregnant** |  |  |  |
| Rural | 5 (83.3) | 2 (10.0) | 2 (10.5) |
| Urban/peri-urban | 1 (16.7) | 18 (90.0) | 17 (89.5) |
|  |  |  |  |
| **Hemoglobin (g/dL): median (IQR)** | 11.5 (10.5-12.2) | 10.0 (8.8-11.0) | 13 (12.0-13.0) |
| **Anaemia: n (%)** | 102 (35.3) | 174 (72.2) | 31 (12.1) |
